# Cystathionine β-synthase T833C/844INS68 polymorphism: a family-based study on mentally retarded children

**DOI:** 10.1186/1744-9081-1-25

**Published:** 2005-12-26

**Authors:** Samikshan Dutta, Swagata Sinha, Anindita Chattopadhyay, Prasanta Kumar Gangopadhyay, Jotideb Mukhopadhyay, Manoranjan Singh, Kanchan Mukhopadhyay

**Affiliations:** 1 Manovikas Biomedical Research and Diagnostic Centre, E.M. Bypass, Kolkata, India; 2 Chittaranjan National Medical College, Kolkata, India; 3 Department of Medicine, IPGMER, S.S.K.M Hospital, Kolkata, India

## Abstract

**Background:**

Cystathionine β-synthase (CBS) mediates conversion of homocysteine to cystathionine and deficiency in enzyme activity may lead to hyperhomocysteinemia/homocystinuria, which are often associated with mental retardation (MR). A large number of polymorphisms have been reported in the *CBS *gene, some of which impair its activity and among these, a T833C polymorphism in *cis *with a 68 bp insertion at 844 in the exon 8 is found to be associated with mild hyperhomocysteinemia in different ethnic groups.

**Methods:**

The present study is aimed at investigating the association between T833C/844ins68 polymorphism and MR. One hundred and ninety MR cases were recruited after psychometric evaluation. Hundred and thirty-eight control subjects, two hundred and sixty-seven parents of MR probands and thirty cardiovascular disorder (CVD) patients were included for comparison. Peripheral blood was collected after obtaining informed written consent. The T833C/844ins68 polymorphism was investigated by PCR amplification of genomic DNA and restriction fragment length polymorphism analysis, followed by statistical analysis.

**Results:**

The genotypic distribution of the polymorphism was within the Hardy-Weinberg equilibrium. A slightly increased genotypic frequency was observed in the Indian control population as compared to other Asian populations. Both haplotype-based haplotype relative risk analysis and transmission disequilibrium test reveled lack of association of the T833C/844ins68 polymorphism with MR; nevertheless, the relative risk calculated was higher (>1) and in a limited number of informative MR families, preferential transmission of the double mutant from heterozygous mothers to the MR probands was noticed (χ^2 ^= 4.00, *P *< 0.05).

**Conclusion:**

This is the first molecular genetic study of *CBS *gene dealing with T833C/844ins68 double mutation in MR subjects. Our preliminary data indicate lack of association between T833C/844ins68 polymorphism with MR. However, higher relative risk and biased transmission of the double mutation from heterozygous mothers to MR probands are indicative of a risk of association between this polymorphism with mental retardation.

## Background

Cystathionine β-synthase (CBS) catalyzes the condensation of serine and homocysteine to form cystathionine and abnormality in CBS activity is manifested in two major clinical conditions, viz. hyperhomocysteinemia and homocystinuria. Insufficiency in CBS activity may lead to hyperhomocysteinemia [1], which is considered to be an independent risk factor for arteriosclerosis [2]. In addition to that, since homocysteine is vasculotoxic as well as neurotoxic, hyperhomocysteinemia predisposes to cardiovascular disorder (CVD) and cognitive dysfunction [3,4]. On the other hand, gross deficiency in CBS activity is associated with homocystinuria, an inborn recessive metabolic disorder [4]. The major pathologic abnormalities associated with homocystinuria include thromboembolism, ectopia lentis, osteoporosis, mental retardation (MR) and other neurological and psychiatric abnormalities [4]. The neurological malfunctioning can be ascribed to the oxidation of excess homocysteine to homocysteic acid, which interacts with the N-methyl-D-aspartate receptor, causing excessive calcium influx and free radical production, thereby leading to neurotoxicity [5]. In addition, MR associated with B12 and folate deficiency could be attributed to the neurotoxic effects of homocysteine since B12 and folate act as co-factors in the homocysteine to methionine remethylation pathway.

The human *CBS *gene, located at 21q22.3 [6], is known to have a large number of mutations, the majority of which are missense in nature [7]. Three different nonsense mutants, as well as some insertion, deletion and splice variants have also been identified, some of which are polymorphic in nature. A transition mutation, T833C generating a BsrI restriction site [8,9] is known to segregate in *cis *with 844ins68 mutation in exon 8 [10] and is reported to be associated with premature occlusive arterial disease [11]. Ethnic heterogeneity of 844ins68 polymorphism is highly prevalent in African, European and North American populations [9,12,13].

Since the T833C/844ins68 double mutation was not detected by Franco et al. [13] and Pepe et al. [14] in their studies on some Asian populations (Japanese, Tharu, Chinese and Indonesian), this allele has been projected by Pepe et al. [14] as a reliable anthropogenetic marker for discriminating between two major human groups – Africans and Asians. Later on, the 844ins68 variant was observed in Han Chinese population with a very low frequency [15]; however, the T833C polymorphism was not studied. In India, study on T833C mutation revealed 4.76% and 9.99% heterozygotes in control and coronary heart disease patients respectively [16]. In the present study, we have looked into the frequency of the double mutant in control population. We have also investigated the occurrence of this double mutant in MR individuals and a small subset of CVD patients, since hyperhomocysteinemia/homocystinuria is often associated with MR as well as CVD [4]. Familial transmission pattern of the double mutation was analyzed, by haplotype-based haplotype relative risk analysis (HHRR) and transmission disequilibrium test (TDT), using heterozygous parents only.

## Methods

### Study design

Our study population consisted of 138 healthy control individuals, 30 CVD patients (average age 53.5 yrs) and 190 MR children (age range 6–8 yrs) along with their parents. The cardiologist (J.M) involved in the study helped in collection of samples from CVD patients. MR cases were selected from the Outpatient Department of Manovikas Kendra Rehabilitation and Research Institute, Kolkata and diagnosed by mental health professionals (child psychiatrist, child psychologist and neurologist) according to the DSM – IV [17] followed by an IQ assessment with Wechsler Intelligence Scale for Children [18]. Both patients and controls were from different parts of India and belonged to no particular ethnic group. Peripheral blood was collected in EDTA, after obtaining informed written consent from control individuals, CVD patients, and parents of MR probands. Genomic DNA was isolated by standard high salt precipitation method [19]. The Human Ethical Committee of the Institute approved the study protocol.

### PCR amplification

**Figure 1:**
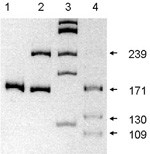
**Representation of double mutation in exon 8 of *CBS *gene: **lane 1 = homozygous genotype without insertion, lane 2 = heterozygous genotype with 68 bp insertion, lane 3 = φX174 HaeIII digest showing bands at 310, 281, 234,194 and 118 respectively, lane 4 = BsrI digestion of heterozygous PCR product (of lane 2), showing complete digestion of the higher allele of 239 bp into 130 bp and 109 bp.

The primer sequence to amplify the 844ins68 polymorphism in the exon 8 and flanking intron 7 of the *CBS *gene were: sense, 5'-GTTGTTAACGGCGGTATTGG-3', and antisense, 5'- GTTGTCTGCTCCGTCTGGTT-3'. PCR amplification was carried out using Perkin Elmer thermal cycler (Gene Amp #2400) in a reaction volume of 20 μL containing 75–100 ng of genomic DNA, 20 pmoles of each primer, 1.0 U Taq polymerase (Bangalore Genei, India), 200 μM dNTP mix, 0.001% gelatin and 10 mM Tris buffer (Bangalore Genei, India) with 50 mM KCl and 1.0 mM MgCl_2_. After an initial denaturation at 95°C for 3 min, amplification was performed at: 35 cycles of denaturation at 94°C for 45 sec, annealing at 58°C for 40 sec and extension at 72°C for 40 sec, followed by a final extension for 5 min at 72°C. PCR products were separated by 12% polyacrylamide gel electrophoresis at 200V for 3 hrs, stained with ethidium bromide and documented in a Gel Doc™ EQ (BIO-RAD). The wild type allele gave 171 bp fragment (A1), and insertion variant gave 239 bp fragment (A2) (Fig. 1).

### Restriction fragment length polymorphism (RFLP) analysis

BsrI digestion was carried out for all samples to detect T833C mutation [9]. PCR products (3 μL) were digested at 65°C for 3 hrs. in a final reaction volume of 25 μL, containing 1 U of BsrI (New England Biolabs; NEB) and NEB Buffer 4. Since this point mutation occurs in *cis *with 844ins68, only the higher allele of 239 bp (A2) was cleaved by BsrI yielding two fragments of 130 bp and 109 bp respectively (Fig 1); wild type allele of 171 bp (A1), however, remained undigested.

### Statistical analysis

Frequency of the T833C/844ins68 polymorphism in the control population and patients was calculated for the Hardy-Weinberg equilibrium. Haplotype-based haplotype relative risk (HHRR) analysis [20] as well as transmission disequilibrium test (TDT) [21] was used to ascertain association between MR and transmission of the double mutation. For TDT, trios with at least one parent having heterozygous genotype were selected.

## Results

Distribution of 844ins68 heterozygote in various control populations studied worldwide is presented in Table I. It was reported earlier that this variant is absent in the Asian population [13,14]. Later studies involving larger sample size revealed the occurrence of 844ins68 variant in the Chinese population [15,25] with a very low frequency of heterozygote as compared to the European and African subjects. We have analyzed the 844ins68 variant in association with the T833C mutation and our data showed a higher rate of occurrence of the double mutant in the Indian control population (7.97%) as compared to the Chinese population.

**Table 1:** Global variation of *CBS *844ins68 polymorphism in control individuals.

Population	2n	Heterozygosity (%)	Reference
US (mixed)	144	11.7	Tsai et al. [9]
Dutch	214	14	Kluijtmans et al. [22]
Japanese	80	0.00	Franco et al. [13]
Chinese	26	0.00	Pepe et al. [14]
Indonesian	98	0.00	Pepe et al. [14]
Ethiopian	108	11.11	Pepe et al. [14]
Spanish	54	25.9	Pepe et al. [14]
Sub-Saharan African	180	66.66	Pepe et al. [14]
German	400	1.5	Linnebank et al. [23]
Chinese (Southern China)	200	5	Zhang & Dai [15]
Italian	820	13.7	Grossmann et al. [24]
Chinese (Northern China)	248	2.97	Li et al. [25]
Indian	276	7.97	Present study

2n = No. of chromosomes

Frequency of the double mutant allele in different types of subjects is presented in Table II. Eleven out of 138 control individuals (n = 276) appeared heterozygous for the mutation. On the contrary, only one person out of 30 CVD patients showed heterozygous genotype. In the MR group, 12 out of 190 MR probands showed presence of the polymorphism and no significant deviation in allele frequencies was noted in the MR probands and their parents (Table II).

**Table 2:** Distribution of *CBS *T833C/844ins68 polymorphism in different groups.

Groups	No of chromosome	No of mutants	Mutant allele frequency ± SE
Control	276	11	0.0398 ± 0.0117
CVD Patients	60	1	0.0166 ± 0.0166
MR probands	380	12	0.0316 ± 0.0089
Parents of probands	534	15	0.028 ± 0.0071

HHRR analysis for the T833C/844ins68 polymorphism showed lack of significant association between MR and transmission of the double mutant (χ^2 ^= 1.714, *P *= 0.1905) (Table III). TDT analysis also revealed lack of association between the T833C/844ins68 polymorphism and MR (χ^2 ^= 1.00, *P *= 0.3173) (Table III). However, transmission from the mother (Table IV), heterozygous for the alleles, revealed that the A2 allele was preferentially transmitted to the MR probands (χ^2 ^= 4.00, *P *= 0.0455), while no significant contribution of the paternal allele was noticed (data not presented).

**Table 3:** HHRR and TDT Analysis for the *CBS *T833C/844ins68 polymorphism in nuclear families with MR probands.

	*Allele*	*Transmitted*	*Non-transmitted*	χ^2^	*P value*	Relative risk (RR)
HHRR analysis	1	259	264	1.714	0.1905	1.346
	2	10	5			
TDT analysis	1	3	6	1.00	0.3173	
	2	6	3			

**Table 4:** Transmission pattern of *CBS *T833C/844ins68 from mother to MR proband.

	Allele	Transmitted	Non-transmitted	χ^2^	*P *value
HHRR analysis (all mothers)	1	176	180	2.0449	0.1527
	2	6	2		
HHRR analysis (heterozygous mothers only)	1	2	6	4.00	0.0455
	2	6	2		

## Discussion

The present finding of 11 out of 138 Indian control individuals with the T833C/844ins68 polymorphism reveal a higher percentage of heterozygotes (7.97%) as compared to the Chinese control population (Table I); but, this value is significantly lower than those quoted for Sub-Saharan population [14]. Two earlier independent studies on Japanese and other Asian populations failed to detect the mutated allele [13,14] and this led to the proposal that this unique double mutation could be used as a reliable anthropogenetic marker for differentiating Asians and Africans. However, the validity of the proposal is questioned by subsequent findings of the 844ins68 polymorphism in different Chinese populations [15,25] and T833C transition mutation in Indian population [16,26]. The present study clearly demonstrates that the T833C/844ins68 polymorphism may not be suitable as an anthropogenetic marker for differentiating Asians and Africans.

Among Indians, presence of T833C heterozygote has been reported in 1 out of 21 (4.76%) [16] and 3 out of 100 (3%) [26] control individuals. However, in both studies the association of 844ins68 mutation with the T833C transition mutation has not been mentioned, while in the present investigation we have observed invariable segregation of these two mutations in *cis*, which is consistent with earlier observations [10,13,14,22].

Hyperhomocysteinemia is hypothesized as an independent risk factor for CVD [22,27] and the 844ins68 variant was previously reported to be associated with premature occlusive arterial disease [11]. A recent article on Chinese congenital heart disease (CHD) patients have shown that the 844ins68 could be a risk factor for CHD, and the insertion especially in mothers could increase the risk in offspring [25]. On the other hand, the findings of Zhang and Dai [15] on adult Chinese patients have suggested that the 844ins68 could provide protection to vascular thromboembolic disease. The mutant allele frequencies among the Indian controls and the CVD patients are 0.0398 and 0.0166 respectively (Table II) and no association was observed between T833C/844ins68 polymorphism and CVD. Heterozygous T833C/844ins68 alleles were observed at higher percentage in Indian CVD patients (3.33%) as compared to Chinese ischemic patients (~1%). The lower value of 3.33% for CVD patients as compared to that of the control (7.97%) seems to support the idea of a possible protective role of the double mutation to vascular thromboembolic disease, as proposed by Zhang and Dai [15]; however, such a conclusion is not called for due to the relatively small CVD sample size in the present study.

While the T833C mutation results in Ile278Thr change in the mutant protein, in the case of T833C/844ins68, the 68 bp insertion results in an alternative splice site generating mostly wild type mRNA with traces of another transcript, detectable only in the nucleus [9,12]. This accounts for the rescue of the transition mutation at 278 in the case of the double mutant [9,10]. However, these observations cannot account for the conflicting reports on the association between cardiovascular disease in different ethnic populations and the mutant allele [28,29]. Case control studies showed that this double mutation might be a neutral polymorphism in the *CBS *gene and splicing of the intron 7 eliminates the mutant allele carrying the T833C [22]. On the other hand, in Northern Europe, the T833C mutation is common in patients presenting clinically with homocystinuria and is associated with a B6-responsive phenotype [22,28].

The *CBS *T833C/844ins68 polymorphism has been reported in homozygous condition, with a very low frequency, from American black artheroslerosis patients [9]. We have failed to observe any subject homozygous for the mutation in our study group, which possibly points towards the deleterious effect of this mutation in homozygous condition.

The present finding of preferential transmission of T833C/844ins68 polymorphism to the MR proband from the heterozygous mother is the first evidence of any association of *CBS *gene with MR, although the association of hyperhomocysteinemia/homocystinuria with psychiatric manifestations including MR is well established [3,4,30]. Among different markers for cobalamine/folate status, plasma homocysteine showed the best association with aging related neuropsychiatric dysfunction [31] and an earlier investigation revealed that the CBS 844ins68 allele frequency was lower in children with high IQ as compared to those with average IQ [32]. In the present investigation, though the HHRR and TDT analysis failed to show any significant transmission of the mutant allele to the MR probands (χ^2 ^= 1.714 and 1.00 respectively), the relative risk (RR = 1.346) calculated showed a risk of MR associated with transmission of the mutant allele. The preferential transmission of T833C/844ins68 polymorphism from the heterozygous mother to the MR proband (χ^2 ^= 4.00, *P *= 0.0455) also supports the above possibility.

## Conclusion

This is the first report on *CBS *T833C/844ins68 polymorphism in association with MR and to the best of our knowledge, this is also the first information on the occurrence of the *CBS *double mutation in the Indian population. Our preliminary data indicate lack of significant association between T833C/844ins68 polymorphism with MR; however, the relative risk calculated and the preferential transmission of the double mutation from informative mothers to MR probands do suggest a risk of association of this polymorphism with MR and warrants further investigation.

## List of Abbreviations used

MR (Mental retardation), CBS (Cystathionine β-synthase), CVD (cardiovascular disorder), HHRR (haplotype-based haplotype relative risk), TDT (transmission disequilibrium test), CHD (congenital heart disease).

## Competing interests

The author(s) declare that they have no competing interests.

## Contributors

SD (JRF, DBT Grant) was responsible for acquisition of data and analysis and drafting the article. SS performed psychometric analysis and clinical diagnosis, intellectual contribution. AC helped in psychological evaluation and IQ determination. PKG was responsible for neurological investigations and intellectual contribution. JG diagnosed the CVD cases. MS revised the manuscript critically. KM was responsible for concept and study design, interpretation of data and revising the article critically. Final manuscript was approved by all authors.
